# Supplementary material for: On the validity of electric brain signal predictions based on population firing rates
Source: PLoS Comput Biol. 2025 Apr 14;21(4):e1012303. doi: 10.1371/journal.pcbi.1012303 (PMC12052147; doi:10.1371/journal.pcbi.1012303)
Supplement: S1 Appendix — Appendix A: Details for the mathematical derivation of the expected error between the ground truth LFP and the population kernel approximation. Appendix B: Details regarding the generation of MIP spike trains and their correlations. Appendix C: Extension to multiple populations. (PDF) [file pcbi.1012303.s001.pdf]

Appendix A. Derivation of error formula

Here we provide details for the derivation of the expected squared error between the ground truth LFP and the population kernel approximation. Inserting the definitions

$$V(\boldsymbol{r},t) = \sum_{j=1}^{N_{\text{pre}}} \left(k_j(\boldsymbol{r},\cdot) * s_j\right)(t) = \sum_{j=1}^{N_{\text{pre}}} \int d\tau\, k_j(\boldsymbol{r},\tau) s_j(t-\tau) \tag{A.1}$$

and

$$\tilde{V}(\boldsymbol{r},t) = \sum_{j=1}^{N_{\text{pre}}} \left(\overline{k}(\boldsymbol{r},\cdot) * s_j\right)(t) = \frac{1}{N_{\text{pre}}} \sum_{i,j=1}^{N_{\text{pre}}} \int d\tau\, k_i(\boldsymbol{r},\tau) s_j(t-\tau) \tag{A.2}$$

into equation (11) in the main text, we obtain

$$\left\langle E^2(\boldsymbol{r})\right\rangle_k = \left\langle \left(\sum_{j=1}^{N_{\text{pre}}} \int d\tau\, k_j(\boldsymbol{r},\tau)\left[s_j(t-\tau)-\nu_j\right] - \frac{1}{N_{\text{pre}}} \sum_{i,j=1}^{N_{\text{pre}}} \int d\tau\, k_i(\boldsymbol{r},\tau)\left[s_j(t-\tau)-\nu_j\right] \right)^2 \right\rangle_{t,k}$$

with firing rates $\nu_j = \left\langle s_j(t-\tau)\right\rangle_t$. Multiplying out the square then yields

$$\left\langle E^2(\boldsymbol{r})\right\rangle_k = \sum_{j,l=1}^{N_{\text{pre}}} \int d\tau \int d\tau' \left\langle k_j(\boldsymbol{r},\tau)k_l(\boldsymbol{r},\tau') - \frac{2}{N_{\text{pre}}} \sum_{i=1}^{N_{\text{pre}}} k_i(\boldsymbol{r},\tau)k_l(\boldsymbol{r},\tau') + \frac{1}{N_{\text{pre}}^2} \sum_{i,k=1}^{N_{\text{pre}}} k_i(\boldsymbol{r},\tau)k_k(\boldsymbol{r},\tau') \right\rangle_k \times \left\langle \left[s_j(t-\tau)-\nu_j\right]\left[s_l(t-\tau')-\nu_l\right]\right\rangle_t .$$

The averages over time $\langle\cdot\rangle_t$ yield the spike-train covariances $c_{jl}(\tau'-\tau) = \left\langle \left[s_j(t-\tau)-\nu_j\right]\left[s_l(t-\tau')-\nu_l\right]\right\rangle_t$, which for stationary spike-train statistics only depends on the relative time between spike trains. For the average over single-cell spike-LFP kernels $\langle\cdot\rangle_k$ one splits the sum $\sum_{jl}$ into a sum over equal indices $\sum_j$ and a sum over unequal indices $\sum_{j\neq l}$ to obtain after some simplifications

$$\left\langle E^2(\boldsymbol{r})\right\rangle_k = (N_{\text{pre}}-1) \int d\tau\, \left(A_k(\boldsymbol{r},\tau) - C_k(\boldsymbol{r},\tau)\right)\left(A_s(\tau)-C_s(\tau)\right)$$

with population-averaged spike-train autocovariance $A_s(\tau) = \frac{1}{N_{\text{pre}}}\sum_{j=1}^{N_{\text{pre}}} \left\langle \left[s_j(t+\tau)-\nu_j\right]\left[s_j(t)-\nu_j\right]\right\rangle_t$, population-averaged spike-train cross-covariance $C_s(\tau) = \frac{1}{N_{\text{pre}}(N_{\text{pre}}-1)}\sum_{j\neq l}^{N_{\text{pre}}} \left\langle \left[s_j(t+\tau)-\nu_j\right]\left[s_l(t)-\nu_l\right]\right\rangle_t$, single-cell spike-LFP kernel autocorrelation $A_k(\boldsymbol{r},\tau) = \int d\tau' \left\langle k_i(\boldsymbol{r},\tau')k_i(\boldsymbol{r},\tau'+\tau)\right\rangle_k$, and single-cell spike-LFP kernel cross-correlation $C_k(\boldsymbol{r},\tau) = \int d\tau' \left\langle k_i(\boldsymbol{r},\tau')k_j(\boldsymbol{r},\tau'+\tau)\right\rangle_k$ for $i \neq j$. In practice, to calculate A_k and C_k, one replaces the expectation value over single-cell spike-LFP kernel statistics by an empirical average that can be measured

$$A_k(\boldsymbol{r},\tau) = \frac{1}{N_{\text{pre}}} \sum_{i=1}^{N_{\text{pre}}} \int d\tau' k_i(\boldsymbol{r},\tau')k_i(\boldsymbol{r},\tau'+\tau)\,,$$

$$C_k(\boldsymbol{r},\tau) = \frac{1}{N_{\text{pre}}(N_{\text{pre}}-1)} \sum_{i\neq j}^{N_{\text{pre}}} \int d\tau' k_i(\boldsymbol{r},\tau')k_j(\boldsymbol{r},\tau'+\tau)\,.$$

Analogous to the calculation above, the variance of the ground truth LFP can be calculated on expectation

$$\left\langle\left\langle[V(\boldsymbol{r},t)-\langle V(\boldsymbol{r},t)\rangle_t]^2\right\rangle_t\right\rangle_k = N_{\text{pre}}\int d\tau\, A_k(\boldsymbol{r},\tau)A_s(\tau)+N_{\text{pre}}(N_{\text{pre}}-1)\int d\tau\, C_k(\boldsymbol{r},\tau)C_s(\tau)\,. \tag{A.3}$$

By expressing A_k and C_k in terms of impulse responses (equation (4) in the main text), we obtain

$$\begin{aligned}
A_k(\boldsymbol{r},\tau) &= \int d\tau'\,\langle k_i(\mathbf{r},\tau')k_i(\mathbf{r},\tau'+\tau)\rangle_k \\
&= \sum_{m,n=1}^{N_{\text{post}}}\int d\tau'\,\langle h_{mi}(\mathbf{r},\tau')h_{ni}(\mathbf{r},\tau'+\tau)\rangle_h \\
&= \sum_{m,n=1}^{K_{\text{out}}}\int d\tau'\,\langle J_{mi}J_{ni}\rangle_J\,\langle \chi_{mi}(\boldsymbol{r},\tau')\chi_{ni}(\boldsymbol{r},\tau'+\tau)\rangle_\chi \\
&= \sum_{m=1}^{K_{\text{out}}}\int d\tau'\,\left\langle J_{mi}^2\right\rangle_J\langle \chi_{mi}(\boldsymbol{r},\tau')\chi_{mi}(\boldsymbol{r},\tau'+\tau)\rangle_\chi \\
&+ \sum_{m\neq n}^{K_{\text{out}}}\int d\tau'\,\langle J_{mi}\rangle_J\langle J_{ni}\rangle_J\langle \chi_{mi}(\boldsymbol{r},\tau')\rangle_\chi\langle \chi_{ni}(\boldsymbol{r},\tau'+\tau)\rangle_\chi \\
&\approx K_{\text{out}}\left(\text{Var}(J)+\text{Mean}(J)^2\right)A_\chi(\boldsymbol{r},\tau)+K_{\text{out}}^2\text{Mean}(J)^2\bar{\chi}^2(\boldsymbol{r},\tau)\,, \\
C_k(\boldsymbol{r},\tau) &= \int d\tau'\,\langle k_i(\mathbf{r},\tau')k_j(\mathbf{r},\tau'+\tau)\rangle_k \\
&= \int d\tau'\,\langle k_i(\mathbf{r},\tau')\rangle_k\langle k_j(\mathbf{r},\tau'+\tau)\rangle_k \\
&\approx K_{\text{out}}^2\text{Mean}(J)^2\bar{\chi}^2(\boldsymbol{r},\tau) \\
A_k(\boldsymbol{r},\tau)-C_k(\boldsymbol{r},\tau) &\approx K_{\text{out}}\left(\text{Var}(J)+\text{Mean}(J)^2\right)A_\chi(\boldsymbol{r},\tau)\,,
\end{aligned}$$

with $\text{Mean}(J)=\langle J_{ki}\rangle_J$ and $\text{Var}(J)=\left\langle J_{ki}^2\right\rangle_J-\langle J_{ki}\rangle_J^2$ the mean and variance of synaptic weights, K_{out} the out-degree of presynaptic neurons, and impulse-response statistics

$$\begin{aligned}
\bar{\chi}^2(\boldsymbol{r},\tau) &= \int d\tau'\,\frac{1}{K_{\text{out}}}\sum_{m=1}^{K_{\text{out}}}\langle\chi_{mi}(\boldsymbol{r},\tau')\rangle_\chi\,\frac{1}{K_{\text{out}}}\sum_{n=1}^{K_{\text{out}}}\langle\chi_{ni}(\boldsymbol{r},\tau'+\tau)\rangle_\chi \\
&\approx \int d\tau'\,\frac{1}{K_{\text{out}}}\sum_{m=1}^{K_{\text{out}}}\frac{1}{N_{\text{pre}}}\sum_{i=1}^{N_{\text{pre}}}\chi_{mi}(\boldsymbol{r},\tau')\frac{1}{K_{\text{out}}}\sum_{n=1}^{K_{\text{out}}}\frac{1}{N_{\text{pre}}}\sum_{i=1}^{N_{\text{pre}}}\chi_{ni}(\boldsymbol{r},\tau'+\tau) \\
A_\chi(\boldsymbol{r},\tau) &= \int d\tau'\,\frac{1}{K_{\text{out}}}\sum_{m=1}^{K_{\text{out}}}\langle\chi_{mi}(\boldsymbol{r},\tau')\chi_{mi}(\boldsymbol{r},\tau'+\tau)\rangle_\chi \\
&\approx \int d\tau'\,\frac{1}{K_{\text{out}}}\sum_{m=1}^{K_{\text{out}}}\frac{1}{N_{\text{pre}}}\sum_{i=1}^{N_{\text{pre}}}\chi_{mi}(\boldsymbol{r},\tau')\chi_{mi}(\boldsymbol{r},\tau'+\tau)\,.
\end{aligned}$$

Appendix B. MIP spike train generation and correlations

Let's consider a homogenous Poisson spike train $m(t)$ ("mother spike train“) of rate ν und independent Poisson spike trains $S_i(t)$ of rate $(1-f)\nu$. We define child spike trains $s_i(t)$ as a superposition of $S_i(t)$ and $m_i(t)$, where $m_i(t)$ is a Poisson process of rate $f\nu$ that consists of a randomly chosen fraction f of spikes from the mother spike train $m(t)$. By definition, each child spike train is then a Poisson process with rate ν and auto-covariance $A_s(\tau) = \nu\delta(\tau)$. Since each spike of the mother spike train is selected with probability f^2 to be copied into $m_i(t)$ and $m_j(t)$, the child spike trains s_i and s_j share a common Poisson spike train of rate $f^2\nu$. The cross-covariance between child spike trains is therefore $C_s(\tau) = f^2\nu\delta(\tau)$, and the correlation coefficient is $c = f^2$.

Since both auto- and cross-covariances of MIP spike trains are proportional to the firing rate, the latter exactly cancels in the relative error of the population kernel approximation (equation (13) in the main text). The absolute error is given by the difference $A_s(\tau)-C_s(\tau) = \nu(1-c)\delta(\tau)$ and therefore rather insensitive to correlations c that are small (Figure 5B, Figure 10B in the main text). For the signal amplitude, cross-covariances are, however, amplified by a factor N_{pre} (equation (A.3)), leading to a strong dependence on c of the signal amplitude (Figure 5A, Figure 10A in the main text) and the relative error (Figure 5C, Figure 10C in the main text).

Appendix C. Extension to multiple populations

In the main text, we derived the error of a population-rate based LFP prediction for a single presynaptic population. We here consider multiple presynaptic populations α. The total extracellular signal then is a linear superposition of the individual populations:

$$V(\boldsymbol{r},t) = \sum_{\alpha} V_\alpha(\boldsymbol{r},t)\,. \tag{C.1}$$

As shown in the main text, each population signal can be approximated by a population-kernel prediction $\tilde{V}_\alpha(\boldsymbol{r},t)$, yielding the overall approximation

$$\tilde{V}(\boldsymbol{r},t) = \sum_{\alpha} \tilde{V}_\alpha(\boldsymbol{r},t)\,. \tag{C.2}$$

The error of this overall approximation is defined as in equation (4.8) in the main text,

$$\begin{aligned}
E^2(\boldsymbol{r}) &= \text{Var}\left[V(\boldsymbol{r},t) - \tilde{V}(\boldsymbol{r},t)\right] \\
&= \sum_{\alpha} \text{Var}\left[V_\alpha(\boldsymbol{r},t) - \tilde{V}_\alpha(\boldsymbol{r},t)\right] + \sum_{\alpha\neq\beta} \text{Cov}\left[V_\alpha(\boldsymbol{r},t) - \tilde{V}_\alpha(\boldsymbol{r},t)\,,\, V_\beta(\boldsymbol{r},t) - \tilde{V}_\beta(\boldsymbol{r},t)\right] \\
&= \sum_{\alpha} E_\alpha^2(\boldsymbol{r}) + \sum_{\alpha\neq\beta} \text{Cov}\left[V_\alpha(\boldsymbol{r},t) - \tilde{V}_\alpha(\boldsymbol{r},t)\,,\, V_\beta(\boldsymbol{r},t) - \tilde{V}_\beta(\boldsymbol{r},t)\right].
\end{aligned} \tag{C.3}$$

It is thus the summed error of the population-kernel predictions of all populations plus a contribution from the covariance of deviations of the population-kernel predictions from the respective ground truth signals. Since different presynaptic populations typically have common postsynaptic targets, the spike-LFP kernels of different presynaptic populations are not independent, leading to a non-vanishing covariance in equation (C.3). Furthermore, spiking activities can be correlated across different presynaptic populations, which also yields a nontrivial contribution to the covariance in equation (C.3). The total error in a multi-population scenario is thus not just the sum of the individual population errors, but depends in a nontrivial manner on the spike-train and spike-LFP kernel correlations across different presynaptic populations.
