# Supplementary material for: On the validity of electric brain signal predictions based on population firing rates
Source: PLoS Comput Biol. 2025 Apr 14;21(4):e1012303. doi: 10.1371/journal.pcbi.1012303 (PMC12052147; doi:10.1371/journal.pcbi.1012303)
Supplement: S1 Fig — The LFP kernels for all tested parameter combinations in Table 1, and the resulting LFP signals (bottom row). The spike trains were from an uncorrelated Poisson process with a firing rate of 10s−1. (PDF) [file pcbi.1012303.s002.pdf]

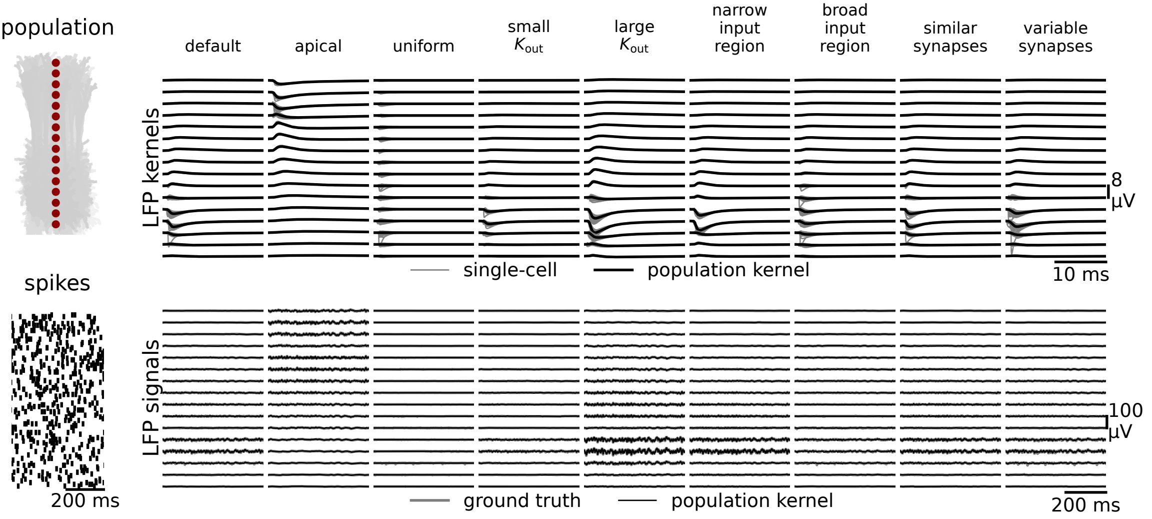
population
default
apical
uniform
small $K_{out}$
large $K_{out}$
narrow input region
broad input region
similar synapses
variable synapses
LFP kernels
8 µV
single-cell
population kernel
10 ms
spikes
LFP signals
100 µV
200 ms
ground truth
population kernel
200 ms
